# Yubitsume: ritualistic self-amputation of proximal digits among the Yakuza

**DOI:** 10.5249/jivr.v6i2.489

**Published:** 2014-07

**Authors:** Anand N. Bosmia, Christoph J. Griessenauer, R. Shane Tubbs

**Affiliations:** ^*a*^Pediatric Neurosurgery, Children’s Hospital, Birmingham, AL, USA.; ^*b*^Division of Neurosurgery, Department of Surgery, University of Alabama at Birmingham, Birmingham, AL, USA.

**Keywords:** Amputation, Digit, Japan

## Abstract

Yubitsume is the ritualistic self-amputation of the proximal digits at the distal interphalangeal joint (DIP) among members of the Japanese mafia, or yakuza. This practice of self-mutilation is done as a sign of apology for making a mistake deemed punishable by higher-ranking members or violating the code of the yakuza. Members of the yakuza may present to emergency departments seeking medical assistance to stop hemorrhage or treat infection at the site of injury following self-amputation or to have the severed portion of the injured finger reattached.

## Introduction

Yubitsume is an act of self-mutilation that has become associated with members of the Japanese mafia, which is also known as the yakuza or boryokudan.^1^ Kaplan and Dubro^2^ write that yubitsume can be voluntary or involuntary and define it as follows: “The ritual act within the yakuza of slicing off the little finger at the joint to atone for a mistake.”The term “yubitsume” translates to “finger-shortening”.^3^ Theauthors review this practice of the yakuza.

## Discussion

**Figure 1:**
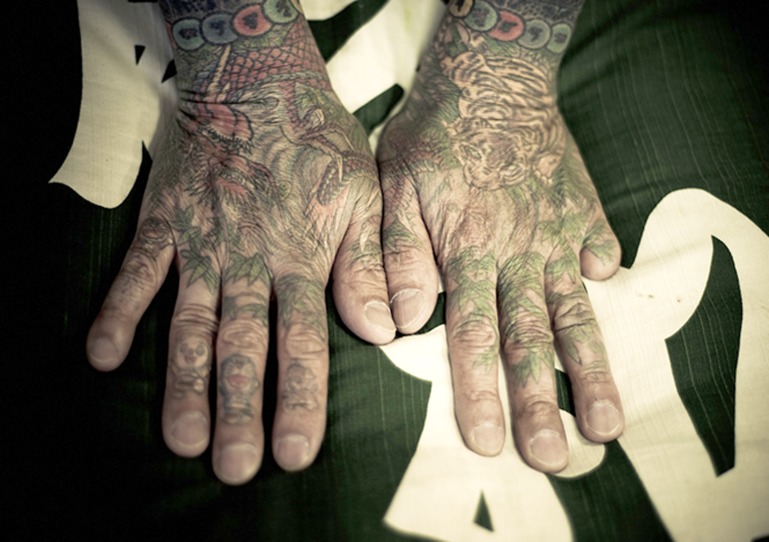
Yakuza member who amputated his small finger on the left hand.^4^ Courtesy of Anton Kusters, who gave permission to publish this photograph.

**Figure 2:**
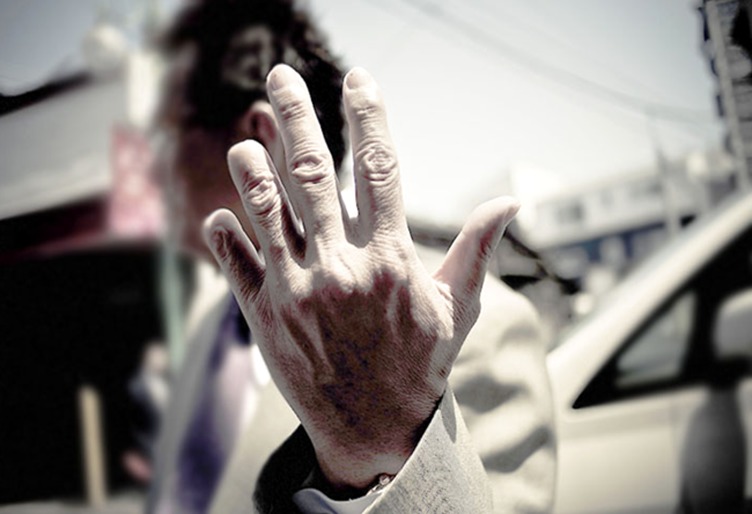
Yakuza member who amputated his small finger on the left hand.^4^ Courtesy of Anton Kusters, who gave permission to publish this photograph.

Physicians in the emergency department may encounter a yakuza member seeking medical assistance following self-amputation (Figure 1-2)^4^ or to have the severed portion of the finger reattached. The member is required to amputate his small finger without any assistance from another member, thus making the ritual more difficult to perform.^3^

A 1993 governmental survey found that 45 percent of modern yakuza members had severed finger joints, and that 15 percent had performed the act at least twice.^2^ Thus, some yakuza members may present with multiple truncated proximal digits. To note, a person who undergoes yubitsume is not necessarily a member of the yakuza. Kirkup^5^ cites the case of a bankrupt Japanese businessman indebted to a yakuza member, who was assisted by a South Korean doctor in 2005 to remove one of his fingers with a hammer and chisel following administration of an anesthetic.

Yubitsume has its origins among Japanese gamblers called bakuto. The bakuto introduced yubitsume as punishment for serious offenses that did not warrant execution or expulsion from the criminal organization to which the offender belonged.^2^ The practice motivated a gambler to pay his debts, for if he failed to do so, he would have to sever a portion of one of his small fingers as an alternative method of payment.^3^ The acute pain of the process was not the only reason yubitsume was feared. Consequences in the long term were also considered. The truncation of the small finger would weaken a gambler’s grip on his sword, thereby putting him at a disadvantage in future sword fights.^3^ In effect, by paying his debts, a gambler safeguarded his adeptness at self-defense.

The pragmatic value of yubitsume to yakuza leaders is that the offending kobun, or soldier, would be viewed as more dependent on protection from his boss, or oyabun.^2^ Furthermore, he would be more vulnerable, as self-amputation would make hand-to-hand combat and handling firearms more difficult. Thus, yubitsume reflects the requirement of members to abide by a strict code of conduct.

Aceti et al^6^ contend that yubitsume normally is not imposed as punishment but instead results from the offender’s own decision to demonstrate his repentance in the hope of avoiding more serious punishment, or is performed to solve a problem or conflict for which the sacrificing party is not responsible. The boss may decide that yubitsume is not satisfactory for atonement, and the member may have to commit seppuku, which is suicide by self-disembowelment,^7^ or be expelled from the yakuza.^3^ When the offender voluntarily amputates his finger to avoid heavier punishment, the amputated finger is called shuniyubi, which means “dead finger”, and when a yakuza member undergoes yubitsume to resolve a conflict, the amputated finger is called ikiyubi, which means “living finger”, and is a symbol of sincere loyalty.^6^ The concept of ikiyubi as presented by Aceti et al implies that the performer of yubitsume is not necessarily an offender, which runs counter to the strongly punitive connotation of the ritual.

There are varying accounts of how the ritual proceeds. Morris^3^ writes: A small piece of clean cloth is placed on a flat surface. The offender places his left hand, palm down, on the cloth and uses a tanto, or sharp knife, to amputate his small finger at the DIP. The severed portion of the small finger is wrapped in the cloth and handed to the head of the offender’s yakuza family, who supervises the event. Rush^8^ writes that the severed portion is wrapped specifically in silk.

Not all descriptions of the ritual include wrapping the finger in cloth or performing the ritual in front of the boss. Kaplan and Dubro^9^ provide the following testimony from a yakuza member who testified against the group: “The actual procedure is to take…what they in Japanese Yakuza call a little silver knife – on a table – and you pull it towards you and bend over and your body weight will snap your finger off…The finger that is severed is put in a small bottle with alcohol and your name is written on it and it is sent to whoever you’re repenting to as a sign that you are sorry.”

Yubitsume is rarely performed now.^10^ The desire of the yakuza to be less conspicuous may have led to the decline of this practice, and the main forms of punishment among the yakuza presently are financial penalties and expulsion from the organization.^11^ Aceti et al^6^ state that yubitsume is less prevalent among younger members of the yakuza, contending that they prefer to pay a fine for their offenses, and also note the shaving of one’s hair, financial penalties, temporary imprisonment, and temporary expulsion as punishments for small offenses. However, heavier punishments exist for more serious offenses. These punishments include yubitsume, rinchi(lynching), hamon (expulsion from the yakuza family), zetsuen(permanent expulsion), and death.^6^ Rankin^11^ contends that yakuza members who cut their fingers today mostly do so involuntarily.

According to interviews with the Japanese National Police Agency, some members use anesthetics to perform yubitsume or go to a hospital to have the severed portion reattached after showing it to their boss.^6^ Abe et al^12^ discuss the case of a 51-year-old yakuza member who amputated his left small finger at the distal phalanx and presented to the emergency department. He had swallowed the amputated portion, and his finger was repaired without reattachment of the amputated portion. The patient said he swallowed the severed portion because he was required to prove that he did not intend to reconstruct his finger. Thus, tolerance of reconstruction of the amputated finger varies among different yakuza families.

The requirement that the ritual be performed solely by the prospective amputee is problematic for individuals less adept at handling knives and who thus sometimes accidentally amputate more than one fingertip or sever the small finger at a location more proximal to the DIP.^5^ These individuals are at greater risk for losing larger amounts of blood. A yakuza member who presents for repair of his amputated digit following yubitsume should be asked if he has been diagnosed with a coagulopathy or has a family history of coagulopathy. A patient with a bleeding disorder is at greater risk for hypovolemic shock. Furthermore, sepsis is a concern in a patient who presents with such an injury and has not attempted to sterilize and dress the wound.
